# Extended Cohort for E-health, Environment and DNA (EXCEED) COVID-19 focus

**DOI:** 10.12688/wellcomeopenres.17437.1

**Published:** 2021-12-16

**Authors:** Paul H. Lee, Anna L. Guyatt, Catherine John, Altaf Ali, Xueyang Wang, Alexander T. Williams, Bo Zhao, Chiara Batini, Catherine Bee, Emma Adams, Carl A. Melbourne, Christopher E. Brightling, Ron Hsu, Jane Bethea, Nicola Reeve, Ioanna Ntalla, Sarah Terry, Manish Pareek, Nigel J. Brunskill, Julian Barwell, Edward J. Hollox, Jose Miola, Susan E. Wallace, David J. Shepherd, Richard Packer, Laura Venn, Louise V. Wain, Robert C. Free, Martin D. Tobin

**Affiliations:** 1 Department of Health Sciences, University of Leicester, Leicester, UK; 2 NIHR Leicester Biomedical Research Centre, University of Leicester, Leicester, UK; 3 Department of Respiratory Sciences, University of Leicester, Leicester, UK; 4 Nottinghamshire Healthcare NHS Foundation Trust, Nottingham, UK; 5 Department of Cardiovascular Sciences, University of Leicester, Leicester, UK; 6 Department of Genetics and Genome Biology, University of Leicester, Leicester, UK; 7 Leicester Law School, University of Leicester, Leicester, UK

**Keywords:** EXCEED, epidemiology, cohort, longitudinal study, genetics, environment, COVID-19, record linkage

## Abstract

**Background:** New data collection in established longitudinal population studies provides an opportunity for studying the risk factors and sequelae of the novel coronavirus disease 2019 (COVID-19), plus the indirect impacts of the COVID-19 pandemic on wellbeing. The Extended Cohort for E-health, Environment and DNA (EXCEED) cohort is a population-based cohort (N>11,000), recruited from 2013 in Leicester, Leicestershire and Rutland. EXCEED includes consent for electronic healthcare record (EHR) linkage, spirometry, genomic data, and questionnaire data.

**Methods:** Between May 2020 and July 2021, a new questionnaire was deployed in EXCEED, which captured COVID-19 symptoms, general physical and mental health, plus socioeconomic and environmental factors during the pandemic. An online system was developed to invite new participants to join EXCEED, with informed consent being provided online. New and existing participants then completed the COVID-19 questionnaire online. A subset of the new questionnaire respondents were invited to participate in COVID-19 serology substudies, using home antibody testing kits.

**Results:** In total, 3,693 participants provided COVID-19 infection status (median age 62.9 (IQR 54.7-69.2), 58.9% female). Trends of monthly incidence proportions of COVID-19 in EXCEED (self-report or symptom-predicted) approximated local and national figures. Regression analysis of 2,768 participants with linked EHR data showed no obvious monotonic relationship between number of chronic diseases (of 16 pre-specified diseases) and COVID-19 infection. There were 2,144 participants with valid results from a kit allowing differentiation between antibodies due to vaccination or infection. Of these, 8.5% had results consistent with previous COVID-19 infection, and 85.9% had evidence of COVID-19 vaccination, but without evidence of infection.

**Conclusions:** Enriching EXCEED with a new COVID-19 questionnaire and serology data may improve understanding of the risk factors, clinical sequelae and broader impacts of the COVID-19 pandemic in the general population. Controlled access to these data for bona fide researchers is via application to the EXCEED study.

## Introduction

In the United Kingdom (UK), the coronavirus disease 2019 (COVID-19) pandemic has highlighted stark pre-existing inequities: infection rates and mortality from COVID-19 are highest in those living in overcrowded housing and areas of deprivation, those working in high-risk occupations (such as key-worker roles,), and in those who have comorbidities ^1^. Individuals from minority ethnic groups are overrepresented amongst these risk groups, which reflects structural discrimination ^1^. There have been far-reaching impacts on people’s livelihoods, with nearly 1 million people projected to be unemployed by the end of 2021 ^1^.

A subset of those infected with COVID-19 experience persistent symptoms (or develop new clinical sequelae) lasting for at least 12 weeks after acute infection, not explained by an alternative diagnosis (‘ post-COVID syndrome/long-COVID’) ^2^. Follow-up of people who have had COVID-19 identified from population samples may help understand: i) who is most at risk of post-COVID syndrome/long-COVID; ii) post-COVID syndrome/long-COVID symptomatology, and iii) what might be the most effective management strategies.

Over 2.2 million people in the UK are members of a cohort study. Enriching pre-existing longitudinal study infrastructure in the UK by combining surveys on the impact of COVID-19, alongside electronic record linkage, plus new recruitment initiatives may therefore be a timely and effective way of collecting relevant data, whilst making use of existing administrative, logistic and governance structures.

We undertook a new wave of recruitment using electronic consent in the Extended Cohort for E-health, Environment and DNA (EXCEED) ^3^, a longitudinal population-based cohort in Leicester, Leicestershire, and Rutland. The availability of linked healthcare record data, baseline spirometry, detailed information on occupation, smoking and lifestyle, and DNA alongside broad informed consent make EXCEED an ideal cohort to study the risk factors for COVID-19 infection and severity, as well as the broader impact of the pandemic on physical, mental and economic wellbeing. This Data Note describes: i) data collected in the EXCEED COVID-19 questionnaire, deployed between 28 ^th^ May 2020 and 6 ^th^ July 2021, and ii) data collected in a subset of COVID-19 questionnaire respondents who agreed to be re-contacted to determine COVID-19 antibody status, collected between 24 ^th^ March 2021 and 22 ^nd^ June 2021. A descriptive summary of respondents is provided, along with monthly incidence proportions of reported or suspected COVID-19, association of COVID-19 with multimorbidity derived from linked electronic health records (EHRs), and summary data of the antibody substudies.

## Materials and methods

### Recruitment

Details of the EXCEED study have been published in the cohort profile paper ^3^. The first wave of EXCEED recruitment (November 2013-December 2018) recruited 10,156 participants aged 30–69 years living in Leicester, Leicestershire, and Rutland. Approximately 95% were recruited via general practice, with other participants recruited from smoking cessation clinics (4.4%), or community-based recruitment, focused on Leicester’s South Asian communities (1.2%). All participants undertook a baseline questionnaire on their lifestyle and health. Around 50% have anthropometry measures and spirometry recorded by trained research professionals. Participants were invited to provide a DNA sample, and 5,214 were genotyped on the Affymetrix UK Biobank array by the Wellcome Trust Centre for Human Genetics (WTCHG), Oxford.

Informed consent for the first wave of recruitment into the EXCEED study was obtained as described in the cohort profile paper ^3^.

In 2020, eligibility for EXCEED was extended to any adult aged 18 or over, living in the East or West Midlands of the UK. After assessing eligibility, participants were directed to the participant information sheet (also publicly available on the EXCEED website), and encouraged to contact the study team with any questions before agreeing to participate in the study. To improve access to the study, the participation information sheet was also translated, and then back-translated for verification by the Centre for Ethnic Health Research at the University of Leicester, and is currently available in Bengali, Gujarati, Punjabi, and Urdu.

Since 2020, recruitment into the study has been via an online, electronic consent process. This was developed in Python, using the Django framework, and provides seamless access to REDCap surveys through a participant profile. The system provides an easy and secure way of recording participants’ consent and tailoring customised questionnaires for different substudies. Eligible participants who wished to participate were asked if they would provide their informed consent via this system. All participants were invited to complete the EXCEED baseline questionnaire after recruitment (this questionnaire was described in the cohort profile paper ^3^).

Recruitment to the COVID-19 antibody substudies is discussed below (see ‘COVID-19 antibody substudies’).

### EXCEED COVID-19 questionnaire (May 2020 – July 2021)

Between 28 ^th^ May 2020 and 6 ^th^ July 2021, existing and newly-recruited EXCEED participants were invited to complete a questionnaire on the physical, psychological, environmental, social, financial and economic impacts of the COVID-19 pandemic. This questionnaire was developed by the Wellcome COVID-19 Questionnaire Steering Group, and included EXCEED Study investigators. The main body of the questionnaire consisted of six ‘core’ sections, all of which were included in EXCEED, alongside selected items from the bank of ‘recommended’ questions ( Box 1). A REDCap implementation of the questionnaire was developed, based on that provided by the Wellcome COVID-19 Questionnaire secretariat (see *Data availability*). Additional data collected in EXCEED included: i) expressions of interest for participation in the antibody substudies, described below, and ii) occupation, automatically mapped via an auto-suggest field to Office for National Statistics (ONS) Standard Occupational Classification (SOC) 2020 codes.

**Box 1. Thematic content of Wellcome COVID-19 questionnaire as adapted for use in EXCEED**

- Section 1: COVID-19 related health questions
  - Physical symptoms
  - Health-seeking behaviour, contacts, shielding, testing and diagnosis
  - Pre-existing physical health conditions and receipt of influenza vaccination
  - Anxiety (GAD-7) ^4^ and depression (PHQ-8, i.e. the first eight questions from PHQ-9) ^5^
  - General self-reported mental and physical wellbeing *
  - Regular medication taken and details *
  - Potential acceptability of a future serology test **
- Section 2: Behaviour change and knowledge
  - Risk mitigation behaviours since the pandemic
  - Changes to lifestyle including risk factors such as smoking and alcohol
  - Changes in communication frequency and mode
  - Changes to children’s lifestyle
  - Changes to children’s education
  - Knowledge of COVID-19 and comprehension of official government guidance
- Section 3: Economic impact
  - Impact on personal finances
  - Food security
  - Changes to job security and employment status, including keyworker status
  - Degree of close contact to others in employment, and access to PPE
  - Most recent occupation, coded according to the Office for National Statistics (ONS) Standard Occupational Classification (SOC) 2020 **
- Section 4: Social impact
  - Changes to living arrangements
  - Isolation and loneliness
  - Household composition and changes to relationship quality
  - Giving and receipt of help as a result of the pandemic
- Section 5: Environmental impact
  - Type of accommodation
  - Problems with damp/mould, vermin
  - Access to outdoor space and nature, natural light
  - Number of rooms in house *
- Section 6: Free text section for capturing other impacts

*Non-core topics selected from bank of Wellcome COVID-19 Questionnaire ‘Recommended’ questions; **EXCEED custom questions

The REDCap implementation of the EXCEED questionnaire was tested for acceptability and ease of use by the EXCEED Patient and Public Involvement Group.

### EXCEED COVID-19 antibody substudies

There were two distinct COVID-19 home antibody testing substudies in EXCEED, which utilised test kits from Fortress and Roche (see below for details of test kits). Initially, 2000 Fortress kits were acquired, and participants who indicated in the COVID-19 questionnaire (completed between May 2020 – July 2021) that they would be interested in participating in antibody substudies were asked to review a Fortress Participant Information Sheet and instructional video. After reviewing these materials, participants were required to complete a home antibody testing consent questionnaire, in which they provided their consent to participate in the Fortress substudy, and in testing via other kits, should they become available in the future. Subsequently, a further test kit (Roche) was made available to EXCEED, and as a result all consenting participants were provided with an additional Roche-focussed Participant Information Sheet and corresponding instructional video.

A total of 2,849 participants agreed to participate in EXCEED’s COVID-19 antibody substudies, and the first 2,000 consenting participants were sent a Fortress home-testing antibody kit ( COVID-19 IgG/IgM Rapid Test Cassette - COVID010) in March 2021. The Fortress test kit is a rapid method for detection of COVID-19 antibodies in 15 minutes, and detects IgM (which will decrease over time and tend to be undetectable by the assay after 6–7 weeks of infection) and IgG antibodies (which persist for a longer period of time) ^6^. Seropositivity is indicated by a red line in the corresponding zone of the test kit. Participants were asked to self-report their test results with the following options: (1) negative (red line next to C, no line next to G or M); (2) IgM positive, IgG negative (red line next to C and M, no line next to G); (3) IgG positive, IgM negative (red line next to C and G, no line next to M); (4) IgG and IgM positive (red line next to C, G, and M); (5) invalid (line next to C is in blue); or (6) can’t tell or not sure. Participants were also asked to upload a photo of their result.

As part of the National Core Studies Serology substudy, EXCEED was given access to additional Roche test kits, which were sent to all 2,849 consented EXCEED participants from April to May 2021. The Roche kit tested for total antibodies against the nucleocapsid protein, which measures response to natural COVID-19 infection, and the spike protein, which measures response to both natural infection and vaccine response ^7^. The participants completed the Roche test at home, then posted the sample to Thriva, and the test results were returned to the research team prior to being shared with participants via their personal EXCEED study profile. Participant queries about their antibody test results were addressed by the research team, and responses to common queries were made available on the EXCEED website.

In addition to the testing kits, participants who were sent a Fortress kit were sent an additional brief questionnaire that collected data on COVID-19 symptoms, vaccination status, and previous COVID-19 testing results.

### Ethical approval

The original EXCEED study was approved by the Leicester Central Research Ethics Committee (Ref: 13/EM/0226). Substantial amendments have been approved by the same Research Ethics Committee for the collection of new data relating to the COVID-19 pandemic, including the COVID-19 questionnaires and antibody testing.

### Statistical analysis

To evaluate the representativeness of the COVID-19 questionnaire respondents, the demographic characteristics of the respondents and the non-respondents were compared using the independent-sample t-test (for continuous variables) or chi-square test (for categorical variables). Socioeconomic status of the EXCEED participants was quantified using the area deprivation level ^3^. The deprivation level of the participants’ living area was obtained from the Ministry of Housing, Communities & Local Government by linking their home address postcode with 2019 Index of Multiple Deprivation (IMD) data, which was grouped into deciles.

A participant was predicted to have COVID-19 in a particular month if they had a predicted probability of >50% of having COVID-19, where the probability equals e ^x^/ (1+e ^x^) for which x = -1.32 - (0.01 × age) + (0.44 × male) + (1.75 × loss of loss of smell or taste) + (0.31 × new persistent cough) + (0.49 × severe fatigue) + (0.39 × decreased appetite), with the symptom value equalling 1 if the participant indicated having the corresponding symptom in the given month and 0 otherwise ^8^, as implemented in the Avon Longitudinal Study of Parents and Children (ALSPAC) COVID-19 questionnaire ^9^. Using the above equation, the monthly symptom-predicted COVID-19 infection incidence proportion was estimated between March 2020 to May 2021 (the incidence proportion for June 2021 was not estimated due to small sample size) and compared with the new cases by date within Leicester, Leicestershire, and Rutland, and the UK as a whole. These nationwide data were obtained from the Gov.uk website using the “new case by publish date” metric from Upper Tier Local Authority of Leicester, Leicestershire, and Rutland (LLR), and the UK as a whole. Monthly incidence proportions for LLR and UK were calculated as the sum of new cases from the first to the last day of the month, divided by the corresponding area population at mid-2019 obtained from Office of National Statistics, as an approximated estimate of the population at risk (Table SAPE22DT15: Mid-2019 Population Estimates for Middle Layer Super Output Areas in England and Wales by Quinary Age Groups and Sex - National Statistics).

COVID-19 infection was defined as either: i) self-reported only (a response of, “Yes, own suspicions”, “Yes, doctor’s suspicion”, or “Yes, diagnosed by positive test” to the question, “Do you think that you have or have had COVID-19?”), or ii) self-reported or symptom-predicted with the above equation, which is a superset of i).

We examined the association between COVID-19 and the number of chronic diseases identified from a participant’s primary care EHR (last date of linkage, 10/11/2018). In total, 16 chronic diseases were analysed, including asthma, cancer, multiple cardiovascular outcomes, chronic kidney disease, chronic obstructive pulmonary disease, diabetes, epilepsy, mental health conditions, osteoporosis, and rheumatoid arthritis (see 3 for details). Number of comorbidities were categorised as 0, 1, 2, or 3 or more. Associations with COVID-19 for each category were calculated using logistic regression and adjusted for age, sex, and area deprivation index, and the adjusted association between COVID-19 and number of diseases was examined using logistic regression by setting the number of diseases as a continuous variable. R 4.1 was used for the statistical analysis.

## Results

Out of the 10,102 EXCEED participants recruited before 28 May 2020, 9,227 consented to share their data though UK Longitudinal Linkage Collaboration (UKLLC) and the current data analysis is based on these individuals. Of these, 2,943 (31.9%) completed the EXCEED COVID-19 questionnaire between May 2020 – July 2021, and linked primary care electronic health records (EHRs) were available for 2,786 out of 2,943 participants (94.7%). Demographic differences between the respondents and non-respondents are shown in Table 1. Respondents were similar to non-respondents in terms of age and sex (62.7 vs 62.9 years, p=0.24, 57% vs 54% female, p=0.19). More respondents self-identified as being of white ethnicity compared to non-respondents (96% vs 91%, p<0.001), and participants living in a less deprived area were more likely to respond (mean decile of IMD 7.30 vs 6.34, p<0.001).

**Table 1.** Demographic characteristics of coronavirus disease 2019 (COVID-19) questionnaire respondents and non-respondents, and new participants.

Variable	Response rate	Non- respondents (n=6,284)	Respondents of existing EXCEED participants (n=2,943)	p-value (respondent vs non- respondents)	New participants (n=750)	p-value (existing participants vs new participants)
Age (mean (SD))		62.94 (8.63)	62.72 (8.15)	0.25	53.92 (15.24)	<2.2 x 10 ^-16^
Sex (frequency (%))				0.19		9.8 x 10 ^-10^
Male	30.5%	2,790 (44.7%)	1,226 (43.2%)		249 (33.2%)	
Female	31.8%	3,451 (55.3%)	1,612 (56.8%)		501 (66.8%)	
missing		43	105		0	
Ethnic group (frequency (%))				<2.2 x 10 ^-16^		<2.2 x 10 ^-16^
White	32.5%	5,512 (90.7%)	2,654 (96.0%)		627 (86.4%)	
Asian/Asian British	14.5%	370 (6.1%)	63 (2.3%)		57 (7.9%)	
Black/African/Caribbean/Black British/Chinese/Mixed/Others *	19.4%	195 (1.3%)	47 (1.7%)		42 (5.8%)	
missing		206	180		24	
Deprivation decile by post code (mean (SD)) (1=most deprived, 10=least deprived)		6.34 (2.88)	7.30 (2.40)	<2.2 x 10 ^-16^	6.34 (2.74)	<2.2 x 10 ^-16^

* Due to small sample size, these groups were combined for confidentiality. SD=standard deviation.

In addition, there were 750 participants who completed in the COVID-19 questionnaire but had not previously been part of the EXCEED study, 311 (41.5%) of whom reside in areas outside Leicester, Leicestershire, and Rutland. Due to the relaxation of minimum age of recruitment to 18, the new participants were younger than the existing participants (53.9 vs 62.7 years, p<0.001). There was a greater proportion of females (67% vs 57%, p<0.001), individuals from minority ethnic backgrounds (14% vs 4%, p<0.001), and a greater degree of deprivation (mean decile of IMD 6.34 vs 7.30, p<0.001) among the new participants compared to the existing participants.

Therefore, there were a total of 3,693 participants (2,943+750) who remained in subsequent analyses. The mean (SD) age of the participants was 60.93 (10.59), 2,113 (58.9%) were female, 3,281 (94.0%) were White, and the mean (SD) deprivation decile was 7.09 (2.50).

A total of 705 participants (19.1%) reported ever having COVID-19 (75 confirmed with a positive test, 47 suspected by a doctor, and 583 suspected by themselves. Note that in England, COVID-19 testing for symptomatic patients in the general public (‘pillar 2’) was not widely available until the second half of May 2020.

**Figure 1.**
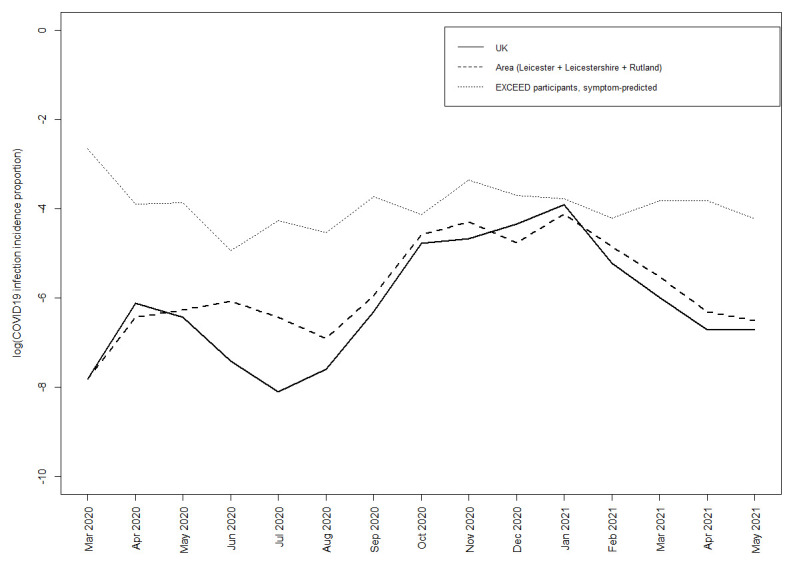
Monthly coronavirus disease 2019 (COVID-19) infection incidence proportions in EXCEED participants predicted by symptom, the UK, and the area of subject recruitment (Leicester, Leicestershire, and Rutland).

Out of the 3693 participants, 458 (12.4%) were predicted to have COVID-19 at any time point between March 2020 and May 2021 based on symptom reporting. Figure 1 shows the incidence of the predicted COVID-19 positive cases across time. During the period between March 2020 to May 2021, the UK incidence proportion and area (Leicester, Leicestershire, and Rutland) incidence proportions were 6.7% and 7.4%, respectively.

Table 2 shows the cross-tabulation of COVID-19 status by self-report and symptom prediction. A strong association was observed between the two measurements, with participants reporting COVID-19 from a doctor’s suspicions being the most likely to be predicted as having COVID-19 by the symptom prediction algorithm.

**Table 2.** Cross-tabulation of coronavirus disease 2019 (COVID-19) status by self-report and symptom prediction.

	Symptom prediction: No COVID-19	Symptom prediction: COVID-19	% predicted as COVID-19
Self-report: No	2769	219	7.3
Self-report: Yes, own suspicions	424	159	27.3
Self-report: Yes, doctor’s suspicion	11	36	76.6
Self-report: Yes, diagnosed by positive test	31	44	58.7

Table 3 shows the association between the number of EHR-linked chronic diseases (until 10/11/2018) and COVID-19 (self-reported between May 2020 and July 2021). There was no clear evidence of a linear association between COVID-19 outcomes and increasing numbers of comorbidities, in either the crude model, or the model adjusted for age, sex and area-level deprivation.

The descriptive results of the antibody levels collected from Fortress (N=1,875) and Roche (N=2,144) kits between March and July 2021 are summarised in Table 4. A total of 1,482 individuals undertook both the Fortress and Roche testing kits. Results from the Fortress kit showed that more than half of the participants had no COVID-19 IgG or IgM antibody. Around 86% of those undertaking the Roche kit had evidence of spike antibodies without nucleocapsid antibodies, suggesting that the COVID-19 antibody response of these individuals was likely induced by vaccination rather than natural infection.

**Table 3.** Association between number of electronic healthcare record (her)-linked chronic diseases (until 10/11/2018) and self-reported or symptom-predicted COVID-19, May 2020 to July 2021 (n=2,768).

Number of EHR-linked chronic diseases ^1^	n	COVID-19 (self-reported) Freq (%)	Adjusted OR (95% CI) ^2^
0	1235	189 (15.3%)	ref
1	937	172 (18.4%)	1.32 (1.04, 1.68)
2	404	66 (16.4%)	1.15 (0.82, 1.59)
3 or more	192	23 (12.0%)	0.93 (0.56, 1.49)
Number of EHR-linked chronic diseases ^1^	n	COVID-19 (self-reported or symptom predicted) Freq (%)	Adjusted OR (95% CI) ^2^
0	1235	255 (20.6%)	ref
1	937	213 (22.7%)	1.19 (0.96, 1.49)
2	404	93 (23.1%)	1.24 (0.93, 1.66)
3 or more	192	33 (17.2%)	0.94 (0.61, 1.42)

^1^ Chronic diseases include asthma, atrial fibrillation, cancer, chronic kidney disease, chronic obstructive pulmonary disease (COPD), coronary heart disease, depression, diabetes, epilepsy, heart failure, hypertension, other mental illnesses (including psychosis, schizophrenia and bipolar affective disorder), osteoporosis, peripheral arterial disease, rheumatoid arthritis, and stroke.

^2^ OR adjusted for age, sex, and area deprivation index. OR=odds ratio; CI=confidence interval.

**Table 4.** Antibody results, March to July 2021.

Fortress (n=1875)	IgM positive	IgM negative
IgG positive	115 (6.1%)	661 (35.3%)
IgG negative	61 (3.3%)	1038 (55.4%)
Roche (n=2144)	Nucleocapsid positive	Nucleocapsid negative
Spike positive	183 (8.5%)	1842 (85.9%)
Spike negative	0 (0%)	119 (5.5%)

## Strengths and limitations of the data

This data note describes new recruitment and data collection in the EXCEED study between May 2020 and July 2021. The updated resource provides a valuable collection of data on COVID-19 infection status, by self-report of infection and/or symptoms, plus data on a subset of participants from two serology substudies, to assess evidence of past infection and/or vaccination. The deployed questionnaire also assessed the impact of the pandemic on physical and mental wellbeing, and socioeconomic factors.

A major strength of the new questionnaire is that the instrument was designed with other cohorts as part of the Wellcome Longitudinal Population Studies Steering Committee, meaning that content is harmonised across cohorts. Similarly, one of the serology substudies was undertaken as part of a cross-cohort initiative. All new data are in addition to the rich data already collected in EXCEED, including baseline questionnaire on health and lifestyle, spirometry, anthropometry, genotype data (EXCEED contributes to the COVID-19 host genetics initiative), and linkage to electronic healthcare records (EHRs), with consent to follow-up for 25 years. EXCEED is also part of the UK Longitudinal Linkage Collaboration: data from collaborating studies may be securely analysed by approved researchers with information from other cohort studies, and linked to whole population health and social records, within a Trusted Research Environment. Details on managed access to EXCEED data are given in the *Data availability* section.

New participants recruited since 2020 were younger on average, and had better representation from minority ethnic groups, the latter being an explicit aim of the study, with targeted communications and translations to facilitate recruitment from minority ethnic groups. New recruitment was online and volunteer-driven, and as different methods of recruitment become possible with different stages of the pandemic, strategies for improved recruitment of specific minority ethnic groups will need to be evaluated, including for males in minority ethnic groups who were under-represented in our study. The majority of the cohort is still of White ethnicity, and we plan to improve the representation of individuals from other ethnic groups; this is crucial given the disproportionate effects of the pandemic on minority groups ^1^. 

As noted in other longitudinal studies ^10^, we observed incomplete response and differential loss-to-follow-up, which may limit generalisability due to selection bias. Around 32% of existing EXCEED participants responded to the COVID-19 questionnaire between May 2020 and July 2021, along with 751 new participants. Those completing the questionnaire (existing and new participants) were also more likely to be from a less deprived background, which is a pattern of attrition observed in other studies ^11^. Respondents may also be different to non-respondents in other unmeasured domains, e.g. technology literacy, given that completion was online.

The data were collected over an extended period, over which pandemic restrictions were changing, and thus considering time of completion of questionnaire in analyses may be useful. Moreover, participants who completed the questionnaire most recently were asked to recall symptoms from the previous 18 months, which will introduce some recall bias, however, linkage to more objective outcomes (e.g. serology) should help correctly classify participants by COVID-19 status. Moreover, the COVID-19 symptom prediction was developed early in the pandemic, and may not remain appropriate as knowledge of symptom clusters produced by different variants emerges.

## Data availability

Data are available via a system of managed access. To apply for access to the data included in this Data Note and other EXCEED data, applicants need to request the EXCEED Data Access Proposal Form via email ( exceed@leicester.ac.uk). We make available data and samples from the study, labelled only with unique codes (no names, addresses, NHS numbers or identifiable data), to researchers approved by the EXCEED Data Access Committee, which is overseen by the EXCEED Independent Scientific Advisory Board. Data access proposals will be reviewed by the EXCEED Data Access Committee and applicants will receive a response within 30 days to advise whether the application has been approved. The EXCEED study encourages requests for collaboration from academic researchers (researchers or employees of an academic institution or the NHS). We are also open to requests for collaboration from commercial organisations, such as companies developing new drug treatments. Potential collaborators must share our scientific goals and be proposing work that fits within the existing study consent.

Please note that costs may apply for use of the resource, for instance if bespoke datasets are required. The Data Access Committee will advise of estimated costs after reviewing proposals.

Please note that text data and any other data deemed potentially disclosive will not be released until they have been coded appropriately.

Several studies, including EXCEED, have deposited de-identified COVID-19 data in a national secure research database, known as the UK Longitudinal Linkage Collaboration (UK LLC). The UKLLC database will enable approved researchers to investigate high-priority COVID-19 research questions and investigate health and wellbeing throughout and beyond the COVID-19 pandemic. Researchers wishing to access this resource can enquire via: www.ukllc.ac.uk.

## References

[1] Marmot M Allen J : COVID-19: exposing and amplifying inequalities. *J Epidemiol Community Health.* 2020;74(9):681–2. 10.1136/jech-2020-214720 32669357 PMC7577092

[2] Whitaker M Elliott J Chadeau-Hyam M : Persistent symptoms following SARS-CoV-2 infection in a random community sample of 508,707 people.Imperial College London Preprint. 2021. Reference Source

[3] John C Reeve NF Free RC : Cohort Profile: Extended Cohort for E-health, Environment and DNA (EXCEED). *Int J Epidemiol.* 2019;48(3):678–9j. 10.1093/ije/dyz073 31062032 PMC6659362

[4] Spitzer RL Kroenke K Williams JBW : A brief measure for assessing generalized anxiety disorder: the GAD-7. *Arch Intern Med.* 2006;166(10):1092–7. 10.1001/archinte.166.10.1092 16717171

[5] Kroenke K Spitzer RL Williams JB : The PHQ-9: validity of a brief depression severity measure. *J Gen Intern Med.* 2001;16(9):606–13. 10.1046/j.1525-1497.2001.016009606.x 11556941 PMC1495268

[6] Jacofsky D Jacofsky EM Jacofsky M : Understanding Antibody Testing for COVID-19. *J Arthroplasty.* 2020;35(7S):S74–S81. 10.1016/j.arth.2020.04.055 32389405 PMC7184973

[7] Houlihan CF Beale R : The complexities of SARS-CoV-2 serology. *Lancet Infect Dis.* 2020;20(12):P1350–P1. 10.1016/S1473-3099(20)30699-X 32979317 PMC7511169

[8] Menni C Valdes AM Freidin MB : Real-time tracking of self-reported symptoms to predict potential COVID-19. *Nat Med.* 2020;26(7):1037–40. 10.1038/s41591-020-0916-2 32393804 PMC7751267

[9] Northstone K Howarth S Smith D : The Avon Longitudinal Study of Parents and Children - A resource for COVID-19 research: Questionnaire data capture April-May 2020 [version 2; peer review: 2 approved]. *Wellcome Open Res.* 2020;5:127. 10.12688/wellcomeopenres.16020.2 33628949 PMC7883314

[10] Fernández-Sanlés A Smith D Clayton GL : Bias from questionnaire invitation and response in COVID-19 research: an example using ALSPAC [version 1; peer review: 1 approved]. *Wellcome Open Res.* 2021;6:184. 10.12688/wellcomeopenres.17041.1 35919505 PMC9294498

[11] Howe LD Tilling K Galobardes B : Loss to follow-up in cohort studies: bias in estimates of socioeconomic inequalities. *Epidemiology.* 2013;24(1):1–9. 10.1097/EDE.0b013e31827623b1 23211345 PMC5102324

