# Supplementary material for: Extended Cohort for E-health, Environment and DNA (EXCEED) COVID-19 focus
Source: Wellcome Open Res. 2021 Dec 16;6:349. [Version 1] doi: 10.12688/wellcomeopenres.17437.1 (PMC11635928; doi:10.12688/wellcomeopenres.17437.1)
Supplement: Peer review history

# Reviewer report: Reviewer response for version 1

**DOI:** 10.21956/wellcomeopenres.19282.r112219

**Published:** 2024-12-10

**Authors:** Ayoung Jeong

**Affiliations:** 1 Swiss Tropical and Public Health Institute, Allschwil, Switzerland; 2 University of Basel, Basel, Switzerland

The Data Note “Extended Cohort for E-health, Environment and DNA (EXCEED) COVID-19 focus” presented the new data collected in the context of the ongoing population-based EXCEED cohort with specific focus on the COVID-19. Given that the pandemic has far-reaching impacts on the population’s wellbeing – physical, mental, and societal wellbeing and beyond, it is crucial to collect relevant data as done in this article. Moreover, by collecting the data in the context of a longitudinal cohort, the new data allows researchers to take advantage of the data that had been collected from the same participants but before the pandemic. Therefore, I believe the data described in this Data Note is timely and meaningful. I found some room for improvement to make the data possibly more useful.

 One of the rationale the authors mentioned in the Introduction is the long-COVID. However, it is not clear what information was collected that can be used to investigate the long-COVID. It could be of interest at least to report if the COVID-19 questionnaire will be repeated in the EXCEED cohort.

 Table 1 demonstrated clearly that the minority ethnic groups are now better represented in the new recruitment. It could have been more informative if the table was extended to report the descriptive statistics of the general population in the study area and in the UK, e.g. the distribution of the ethnicity.

 The new data collection included the antibody test results using two different kits. It could be informative to report the time interval between the two, in case the same participants used both kits (N=1482).

 In the Statistical analysis section, the authors mentioned a logistic regression using the number of diseases as a continuous variable. However, I cannot find the corresponding results. Table 3 reported association analysis results, but it does not appear to be from a logistic regression using the number of EHR-linked chronic diseases as a continuous variable.

 Based on the results in Table 3, the authors reported “no clear evidence of a linear association between COVID-19 outcomes and increasing numbers of comorbidities”. This should not discourage future research towards the effects of comorbidities on the COVID-19 outcomes; in particular, the current analysis does not investigate the potential effect modification by comorbidities.

**Minor comments:**

- The numbers in the first paragraph of the Results section deviate from Table 1 (e.g. p-value 0.24 vs 0.25; female 54% vs 55%).
- Figure 1 could have been more informative if the y-axis was in the original scale.
- Table 2 contains a typo in the title: … (her) should be replaced by … (EHR).

Are sufficient details of methods and materials provided to allow replication by others?

Yes

Is the rationale for creating the dataset(s) clearly described?

Yes

Are the datasets clearly presented in a useable and accessible format?

Yes

Are the protocols appropriate and is the work technically sound?

Yes

Reviewer Expertise:

Cohort studies; Environmental epidemiology; Subjective wellbeing

I confirm that I have read this submission and believe that I have an appropriate level of expertise to confirm that it is of an acceptable scientific standard.

# Reviewer report: Reviewer response for version 1

**DOI:** 10.21956/wellcomeopenres.19282.r53355

**Published:** 2023-04-13

**Authors:** Miryam Lora-Loza

**Affiliations:** 1 Universidad César Vallejo, Trujillo, Peru

The article entitled "Extended Cohort for Electronic Health, Environment and DNA (EXCEED) COVID-19 Approach" being an epidemiological study of the Prospective Analytical Cohort presents an appropriate title and the number of words required according to the format of the journal.

 In reviewing the abstract, it is clear that the purpose of the data note needs to be more pertinently stated, using the structure in which the verb plus content should be included. The work in the first instance pursues the proposed objective: "to study the risk factors and sequelae of the new coronavirus disease 2019 (COVID-19), in addition to the indirect impacts of the COVID-19 pandemic on well-being", which is consistent with the title.

 Regarding the introduction, it is suggested that the nature of the note should be indicated in the objectives since it declares a data note composed of a preliminary analysis of the database. Data using a proprietary instrument, in such a way that the objective declared in summary coincides with or declared in the content of the study. Therefore, it is suggested to mention the general objective of the note. It should also be noted that the justification that supports the work is not appreciated.

 Regarding materials and methods. It is necessary to clarify that, from a total of approximately 10,102 participants, from whom they have collected information to write this data note, it is suggested to focus on the sampling carried out, as well as its limitations, also to include what were the criteria sample selection. It is also essential that the authors establish the eligibility criteria in the inclusion-exclusion environment. The use of Python is appreciated and correct for dealing with big data, but when enunciating tests such as chi square and "t" of student, and at the same time Odds Ratio, it is suggested to be more explicit in the use of each of these tests and that these are asserted in the result.

 Regarding the instrument, a factorial character is glimpsed where the psychological, behavioral, economic, social, environmental and physiological aspects are capable of clarifying an equation that projects the probability of acquiring a COVID-19 infection or suffering from comorbidity, and how it is even in construction it is convenient to declare it since this document is a Data Note, I also suggest strengthening the citations that are very important in the methodological and ethical aspect (insert the link of the database).

 In relation to the results: The results show a coherent way of representing sensitivity to antibodies, comorbidities and intervening physical factors, it is suggested that as it is a Data Note it is declared if the factors will be included in a future document. Behavioral, environmental, social, or economic factors that may be a baseline risk factor for the data; only the infectious factor is visualized.

 Regarding the reference. The use of bullets and at the same time of numbers is observed, being a factor that must respond only to the Vancouver standard. Likewise, it is suggested that, if the Vancouver standard is used, it is necessary to standardize the criteria to avoid placing links, as it appears in it. Therefore, it is suggested that these links go to the bibliographical reference and be cited under the Vancouver standard.

Are sufficient details of methods and materials provided to allow replication by others?

Yes

Is the rationale for creating the dataset(s) clearly described?

Yes

Are the datasets clearly presented in a useable and accessible format?

Yes

Are the protocols appropriate and is the work technically sound?

Yes

Reviewer Expertise:

Public health

I confirm that I have read this submission and believe that I have an appropriate level of expertise to confirm that it is of an acceptable scientific standard.
